# Combined Economic Emission Dispatch of Microgrid with the Incorporation of Renewable Energy Sources Using Improved Mayfly Optimization Algorithm

**DOI:** 10.1155/2022/6461690

**Published:** 2022-04-18

**Authors:** Karthik Nagarajan, Arul Rajagopalan, S. Angalaeswari, L. Natrayan, Wubishet Degife Mammo

**Affiliations:** ^1^Department of Electrical & Electronics Engineering, Hindustan Institute of Technology & Science, Chennai, Tamil Nadu, India; ^2^School of Electrical Engineering, Vellore Institute of Technology, Chennai 600127, Tamil Nadu, India; ^3^Department of Mechanical Engineering, Saveetha School of Engineering, SIMATS, Chennai 602105, Tamil Nadu, India; ^4^Mechanical Engineering Department, Wollo University, Kombolcha Institute of Technology, Kombolcha, South Wollo–208, Amhara, Ethiopia

## Abstract

Electricity can be provided to small-scale communities like commercial areas and villages through microgrid, one of the small-scale, advanced, and independent electricity systems out of the grid. Microgrid is an appropriate choice for specific purposes reducing emission and generation cost and increasing efficiency, reliability, and the utilization of renewable energy sources. The main objective of this paper is to elucidate the combined economic emission dispatch CEED problem in the microgrid to attain optimal generation cost. A combined cost optimization approach is examined to minimize operational cost and emission levels while satisfying the load demand of the microgrid. With this background, the authors proposed a novel improved mayfly algorithm incorporating Levy flight to resolve the combined economic emission dispatch problem encountered in microgrids. The islanded mode microgrid test system considered in this study comprises thermal power, solar-powered, and wind power generating units. The simulation results were considered for 24 hours with varying power demands. The minimization of total cost and emission is attained for four different scenarios. Optimization results obtained for all scenarios using IMA give a comparatively better reduction in system cost than MA and other optimization algorithms considered revealing the efficacy of IMA taken for comparison with the same data. The proposed IMA algorithm can solve the CEED problem in a grid-connected microgrid.

## 1. Introduction

Microgrid is one of the advanced small-scale centralized electricity systems and it usually contains energy storage resources, Distributed Generation (DG) units, and loads. Microgrids are generally designed and installed nearby energy consumers in a confined community [1]. But there has been a drastic increase in recent years regarding installing renewable energy sources in microgrids, owing to environmental advantages and low cost compared to its counterparts [2]. Microgrid meets different load requirements in residential, commercial, agriculture, and industrial sectors [3]. Microgrids can function under two different modes: grid-connected and islanded modes. In the former mode, the microgrid and main grid are connected, while in the latter the microgrid is isolated from the main grid during an emergency outbreak. It continues its power delivery functions to local loads as usual [4]. There are loads of advantages present in using microgrid reduction of carbon emission and generation cost, thanks to renewable energy sources, and increased reliability and power quality, etc. [5].

Optimal operations and effective planning of electric power generation systems are the two most crucial elements in electric industries. Controlling and operating power systems, cost-efficient load dispatch (Economic Load Dispatch, i.e., ELD) related problems are too much concerning to address [6]. Power system optimization problems that employ ELD are useful in identifying the most suitable, cheap, and seamless operations with the regulation of outputs produced by different power generation units that meet the load demands. ELD has a primary aim to mitigate the overall cost incurred upon power generation without compromising or producing any constraints [7]. ELD determines active power output generated by different power generation systems to attain the objective functions and simultaneously overcome many problems [8]. It is important to develop novel power management algorithms to translate the microgrid as a viable and happening choice compared to traditional power systems [9]. The mitigation of both power generation costs and the emission of environmental pollutants is the sole aim of utility operators. For these goals, two contradictory objectives must be considered. Combined economic emission dispatch (CEED) is utilized to mitigate emission levels from all generating units and costs incurred by the operating units [9].

CEED problem has been discussed earlier by different authors who proposed several optimization techniques to overcome the issue [10]. In [10], the researchers proposed a balanced trade-off method to resolve the ECED (Environment Constrained Economic Dispatch) problem. The study conducted a first-of-its-kind comparative analysis of three methods: Fractional Programming (FP), ECED, and price penalty factor (PPF) to overcome the CEED problem. Chimp Optimization Algorithm (ChOA) was proposed to address the optimal design of microgrid which comprises PV panels, wind turbines, and battery storage systems [11]. Optimization results had been compared with Improved Grey Wolf Optimizer (IGWO) and Grey Wolf Optimizer (GWO). Optimal sizing of photovoltaic cell and solar water heater by considering environmental parameters and fuel-saving was carried out in [12]. Energy and economic analysis of solar energy-based cogeneration system for a building in Saveh City were studied. The researchers simulated the model in a 3-Unit dynamic test system incorporating renewable energy sources. In the study conducted by Alamoush [13], Bernstein-search differential evolution (BSDE) algorithm was proposed to resolve the Dynamic Combined Heat and Power Economic emission Dispatch (DCHPED) generation problem in microgrid comprised of renewable energy sources, fixed nondeferrable and deferrable loads, fossil-fuel combined heat and power units, and thermal energy storage devices. In [14], whale optimization algorithm (WOA) has been applied to carry out the combined economic emission dispatch problem. The simulation was performed by considering four different load sharing scenarios among the distributed energy resources. The researchers also conducted ANOVA and Wilcoxon signed-rank tests to validate the supreme characteristic of WOA. In [15], Stochastic Fractal Search (SFS) algorithm was applied to resolve multiobjective economic emission dispatch problems that arise in combined heat and power (CHP) generation. This study was conducted in large microgrids in which solar-powered generating units, wind power units, and fossil-fuel-powered generating units were installed.

Collective Neuro Dynamic Optimization (CNO) method was proposed in the earlier study. In this study, the authors combined a heuristic approach and projection neural network (PNN) to optimize the scheduling of an electrical microgrid containing ten thermal generators and mitigate the costs incurred upon emission and generation [16]. A mixed-integer nonlinear programming formulation was proposed in [17] to dispatch the distributed generators cohesively. Further, the model was also aimed at fulfilling the water demands and the building's thermal energy requirements in a standalone water energy microgrid.

In [18], Giza Pyramids Construction (GPC) was proposed to implement the optimal design of an isolated microgrid. Net present cost (NPC), Levelized Cost of Energy (LCOE), loss of power supply probability, and availability index were considered objective functions. Modified adaptive accelerated particle swarm optimization (MAACPSO) algorithm was proposed to investigate the grid-tied PV systems reliability [19]. This study focused on the probability analysis and reliability assessment of the components of grid-tied PV systems through IEEE 24 bus integrated with PV system with four different case studies. A first-of-its-kind Sequential Optimization Strategy (SOS) was formulated in the study conducted earlier to allocate active and reactive power to Dispatchable Distributed Generator (DDG) units in an optimal manner. These DDG units are installed in a droop-controlled islanded AC microgrid [20]. The research proposed improved Quantum Particle Swarm Optimization (QPSO) earlier [21] to address the Short-Term Economic Environmental Dispatch (EED) problem in a microgrid. Recently, a multiobjective seeker optimization algorithm has been proposed to analyze the influence of charging and discharging behaviour of electric vehicles and demand side response resources on the economic functioning of PV-connected microgrid systems. The model considered three objectives: power fluctuation between microgrid and main grid, comprehensive operating cost of the microgrid, and utilization rate of photovoltaic energy [22].

In [23], the researchers proposed a mathematical optimization approach to achieve an optimal operation upon economic dispatch in DC microgrid. To allot the schedules for unit commitment and achieve economic dispatch in microgrid, the study conducted earlier [24] proposed an enhanced real-coded genetic algorithm in the enhanced mixed-integer linear programming (MILP) based method. The authors proposed a stochastic model in [25] to manage CHP-based microgrids optimally. The study took economic, reliability, and environmental aspects into consideration. Nonconvex and nonlinear stochastic problems are used to resolve complexity. The Exchange Market Algorithm (EMA) was proposed in a study. This study considered three contradictory objectives through a weighted sum approach to resolving the multiobjective problem as a single-objective problem.

A multiobjective optimal dispatch model was proposed in [26] for a grid-connected microgrid. In this study, the authors considered reducing environmental protection costs and the generation cost of the microgrid with the incorporation of an enhanced PSO algorithm. In [27], a genetic algorithm is applied to optimize the focal area of the parabolic trough concentration photovoltaic/thermal system. The objective function was considered a combination of electrical efficiency and thermal efficiency. A parabolic trough concentrating photovoltaic thermal (CPVT) was utilized to afford the energy required for a residential building [28]. CPVT was used as a source of heat and cooling and electrical energy for the building and simulation was carried out using TRNSYS software. Samy et al. addressed the problem of power outages in distant districts by taking advantage of the available renewable energy resources in the contiguous environment [29]. Hybrid Firefly and Harmony Search optimization technique (HFA/HS) was implemented to improve the net present cost of the proposed hybrid system which comprises photovoltaic (PV), wind turbine (WT), and fuel cell (FC). MINLP, a novel optimization model, was proposed in [30] to resolve the economic dispatch problem of a microgrid which contains numerous units of wind turbines (WTs), heat-only units, traditional power generators, photovoltaic (PV) systems, CHP units, and battery storage systems under certain uncertainties. In the study conducted earlier [31], MBGSA (Memory-Based Gravitational Search Algorithm) was proposed to overcome the ELD problem. In one of the research investigations [32], a novel Multiobjective Virus Colony Search (MOVCS) was proposed to elucidate the multiobjective dynamic economic emission dispatch (DEED) problem. This model aims to mitigate the emissions produced by fossil-fuel power generators and simultaneously reduce the cost incurred upon wind-thermal electrical energy costs. In [33], HOMER software is used to model and simulate a wind-solar hybrid system independent of the national grid in the northwest of Iran. A multiobjective particle swarm optimization technique is proposed to optimize the sizing of a green energy system connected to a randomly disrupted grid [34]. The energy cost for evaluating hybrid system economies, the loss of probability of power supply (LPSP) for reliability assessments, and the System Surplus Energy Rates (SSER) were considered objective functions for evaluating hybrid system compatibility and efficiency. A sustainable energy distribution configuration for microgrids integrated into the national grid using back-to-back converters in a renewable power system was examined in [35]. Different scenarios of several sustainability schemes of power management in microgrids were analyzed.

SGEO (Social Group Entropy Optimization) technique was proposed in [36] to resolve Fuel Constrained Dynamic Economic Dispatch (FCDED) with Demand Side Management (DSM). The technique combined the pumped hydrostorage plant with renewable energy sources. This research used a stochastic fractal search algorithm to overcome the biobjective combined heat and power economic dispatch (CHPED) problem [37].

In [38], mayfly algorithm (MA) was proposed by Dr. Konstantinos Zervoudakis in 2021. In this paper, an improved mayfly optimization algorithm was investigated with the help of a microgrid model under varying scenarios. The results were contrasted against recent state-of-the-art algorithms that also employed the same microgrid model.

The contributions of the current research paper are summarized herewith.

- An improved version of the mayfly optimization algorithm incorporating Levy flight is proposed to elucidate the microgrid test system's CEED problem.
- An improved mayfly optimization algorithm is proposed in addition to Levy flight to overcome the CEED problem encountered in microgrid test system.
- Levy flight has been leveraged in this study since it possesses huge advantages in not engaging local optimal. An optimal trade-off is provided by the proposed algorithm between exploration and exploitation phases.
- The authors validated the supremacy of the proposed algorithm in terms of resolving the CEED problem under two different objective functions that involve advanced energy sources.
- Compared with existing population-based optimization tools such as PSO and GA, only a few control parameters exist in IMA. This feature helps in making it the best optimization procedure. IMA-based CEED was authenticated as a unique and robust technique since it incurred less total generation cost than the solution even after conducting multiple random trials.

## 2. Mathematical Formulation of CEED for Microgrid

### 2.1. Combined Economic Emission Dispatch (CEED)

The simultaneous mitigation of economic and environmental dispatch objective functions remains the primary objective for the CEED problem in the microgrid. In other terms, the total fuel cost must be reduced, while at the same time the emission levels should also be mitigated without compromising the constraints. So, it is suggested to formulate CEED as a single optimization problem as given herewith [9].

(1) $$\begin{array}{c} \text{minTC}=\overset{\text{NG}}{\underset{i=1}{\sum }}\left\{F_{i}\left(P_{\text{Gi}}\right),E_{i}\left(P_{\text{Gi}}\right)\right\}. \end{array}$$

Here, TC denotes the total operating cost that should be minimized. The fuel cost of the *i*^th^ generator is represented as *F*_*i*_(*P*_Gi_),   the emission level of the *i*^th^ generator is represented as *E*_*i*_(*P*_Gi_),  *P*_Gi_ denotes the *i*^th^ generating unit's output power, and finally, NG corresponds to the whole generating unit count.

#### 2.1.1. Minimization of Fuel Cost

In general, the fuel cost function is denoted through the quadratic equation given below [9]:

(2) $$\begin{array}{c} F_{t}=\overset{\text{NG}}{\underset{i=1}{\sum }}F_{i}\left(P_{\text{Gi}}\right)=\overset{\text{NG}}{\underset{i=1}{\sum }}\left(x_{i}+y_{i}P_{\text{Gi}}+z_{i}P_{\text{Gi}}^{2}\right). \end{array}$$

Here, *F*_*t*_ corresponds to overall fuel cost incurred in terms of $ and *x*_*i*_($/*h*), *y*_*i*_($/MWh), and *z*_*i*_($/MW^2^h) correspond to the cost coefficients of *i*^th^ generating unit.

#### 2.1.2. Minimization of Emission

When fossil fuels are used, the generators emit different sorts of pollutants. So, pollution mitigation forms the primary goal of most power system operations. Equation (3) denotes the expression for total emission from [5, 9]:

(3) $$\begin{array}{c} E_{t}=\overset{\text{NG}}{\underset{i=1}{\sum }}E_{i}\left(P_{\text{Gi}}\right)=\overset{\text{NG}}{\underset{i=1}{\sum }}\left(\alpha_{i}+\beta_{i}P_{\text{Gi}}+\gamma_{i}P_{\text{Gi}}^{2}\right). \end{array}$$

Here, *E*_*t*_ denotes the overall emission and *α*_*i*_ (kg/h), *β*_*i*_(kg/(MWh)), *γ*_*i*_(kg/(MW^2^h)) correspond to the *i*^th^ generating unit's emission coefficients.

#### 2.1.3. Total Generation Cost of CEED Problem

It is possible to convert the dual-objective optimization problem, focusing emission, and fuel cost, into a single-objective optimization problem with the induction of PPF (price penalty factor) as given earlier [9]:

(4) $$\begin{array}{c} \text{minTC}=F_{t}+\Lambda \times E_{t}. \end{array}$$

Here, Λ denotes the price penalty factor (PPF), which is calculated as a ratio between fuel cost and the emission of the corresponding generating unit ($/kg). PPFs are of different types, while in the current study, the authors use min-max types sourced from [5, 9] for comparison. Following is the equation for min-max type [9]:

(5) $$\begin{array}{c} \Lambda_{i}=\frac{F_{t}\left(P_{\text{Gi}}^{\text{min}}\right)}{E_{t}\left(P_{\text{Gi}}^{\text{max}}\right)},\;i=1,2,\ldots ,\text{NG}, \end{array}$$

where *P*_Gi_^max^ and *P*_Gi_^min^ Here, Λ denotes the ratio between maximum fuel cost and maximum emission of the corresponding generator in $/kg [9]. The maximum and minimum output power of the generator combines emission with fuel cost. Afterwards, TC corresponds to the total operating cost of $.

The following is the list of steps to be followed to determine the price penalty factor for a specific load demand [39].

- The ratio between minimum fuel cost and the maximum emission of every generating unit should be determined.
- Price Penalty Factor values are sorted out in ascending order.
- The maximum capacity of every unit (*P*_Gi_^max^) one is added at a time that starts from the lowest Λ_*i*_, until ∑*P*_Gi_^max^ ≥ *P*_*D*_.
- Then, Λ_*i*_, which has an association with the lowest unit in this process, remains the tentative PPF value (Λ) for the load under consideration.

So, a modified PPF (Λ) is utilized to arrive at the exact value for specific load demand based on the interpolation of Λ values corresponding to their load demand values.

### 2.2. Cost Functions of Renewable Energy Sources

Across the globe, renewable energy sources are the foremost choice of transmission when energy is produced, compared to traditional generators. In such a scenario, solar and wind power can be denoted as negative loads and can be used to mitigate the total load demand in the system [9]. However, the economic dispatch solution is considered a base to distribute the rest of the load demands on traditional generators. In the current study, the CEED solution for the microgrid takes cost functions of wind and solar-powered generating units into account. From [9], the input data for cost and emission coefficients are considered.

#### 2.2.1. Cost Function of Wind Power Generating Unit

Current economic analysis is conducted for wind-based power generation and the specific cost can be determined with inputs, operation, maintenance, and equipment costs. This cost function is expressed as per [5, 9]:

(6) $$\begin{array}{c} C_{w}\left(P_{w}\right)=\left(\frac{r}{\left[1-{\left(1+r\right)}^{-N}\right]}l^{p}+O^{E}\right)P_{w}. \end{array}$$

Here, *P*_*w*_ denotes the wind power produced in terms of kW, *r* corresponds to the interest rate, *a* denotes the Annuitization coefficient, *N* corresponds to lifetime investment in terms of years, and *l*^*p*^ and *O*^*E*^ correspond to the costs incurred upon investment per unit installed power ($/kW) and operating and maintenance costs per unit installed power ($/*kW*), respectively.

The 24-hour data for the wind power generating unit is considered from [5]. The parameters required for wind power cost function are chosen from [5, 9].

#### 2.2.2. Cost Function of Solar Power Generating Unit

Similar to wind power, solar-powered generating unit's cost function is expressed as in [5, 9]:

(7) $$\begin{array}{c} C_{s}\left(P_{s}\right)=\left(\frac{r}{\left[1-{\left(1+r\right)}^{-N}\right]}l^{p}+O^{E}\right)P_{s}, \end{array}$$

where *P*_*s*_ is the output power from the solar-powered generating unit, *r* corresponds to interest rate, *N* denotes lifetime investment in terms of years, and *l*^*p*^ and *O*^*E*^ correspond to investment costs made upon per unit installed power ($/kW) and operating and maintenance costs per unit installed power ($/kW), respectively.

The 24-hour data of the solar power generating unit is considered [5]. The parameters required for solar power cost function are chosen from [5, 9].

### 2.3. Total Cost of CEED for Microgrid

The equation for the total CEED cost for the microgrid is shown below. This value gets minimized based on the cost functions regarding wind and solar power [9].

(8) $$\begin{array}{c} C_{T}=F_{t}+\Lambda \times E_{t}+\left(\frac{r}{\left[1-{\left(1+r\right)}^{-N}\right]}l^{p}+O^{E}\right)P_{w}+\left(\frac{r}{\left[1-{\left(1+r\right)}^{-N}\right]}l^{p}+O^{E}\right)P_{s}. \end{array}$$

### 2.4. Constraints

The researcher considered both generator capacity and power balance constraints to contrast with existing optimization algorithms.

#### 2.4.1. Islanded Mode of Microgrid

The current study considered islanded mode microgrid to compare with the optimization results achieved in [5, 9]. There is no trade-off for the power between the main grid and the microgrid in this mode. So, the microgrid needs to fulfil the local or confined community load demands.

#### 2.4.2. Power Balance Constraint

The load demand must be equal to that of the total power generation [9].

(9) $$\begin{array}{c} \overset{\text{NG}}{\underset{i=1}{\sum }}P_{\text{Gi}}+P_{w}+P_{s}=P_{D}. \end{array}$$

Here, *P*_*D*_ corresponds to the total load demand.

#### 2.4.3. Generation Capacity Constraints

Every generating unit's output power gets flanked by both lower and upper bounds [9].

(10) $$\begin{array}{c} P_{\text{Gi}}^{\text{min}}\leq P_{\text{Gi}}\leq P_{\text{Gi}}^{\text{max}}. \end{array}$$

Here, *P*_Gi_^min^ and *P*_Gi_^max^ correspond to the minimum and maximum output powers of the *i*^th^ generating unit correspondingly.

## 3. Improved Mayfly Algorithm

**Figure 1:**
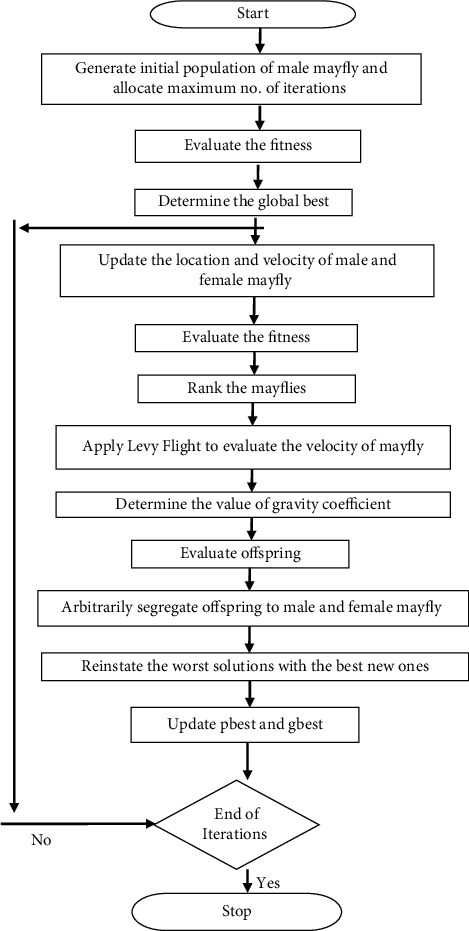
Flowchart of the proposed improved mayfly optimization algorithm.

Mayfly algorithm takes its inspiration from the social behaviour of mayflies, especially how they mate with each other [38]. It is assumed that mayflies are instantly considered adults as soon as the eggs are hatched. Leaving beside the period of their life, only the fittest mayflies tend to survive. Each mayfly has a position in search space that corresponds to a solution that overcomes the problem. RAND functions are utilized in conventional mayfly algorithm to produce novel variables that lead to local optimal. To increase MA's searching ability and create an optimal solution, the researchers integrated MA with Levy flight. If a Levy flight-based approach is utilized for system identification, it achieves rapid convergence and does not entail derivative information [40], attributed to stochastic random search, in line with the Levy flight concept [41]. Levy flight contributes heavily to increasing the optimal solution's local search avoidance and local trapping [42]. The flowchart of the proposed IMA algorithm is shown in Figure 1.

The steps required for the proposed mayfly optimization algorithm works are described as follows:

**Step 1 .**

Two mayfly sets, each representing a male and a female population, should be generated randomly. Then, every mayfly is arbitrarily placed in problem space as a candidate solution which is denoted by a *d*-dimensional vector *P*_Gi_=(*P*_G1_,…,  *P*_Gd_). Then the performance is assessed based on the predefined objective function *f*(*C*_*T*_(*P*_Gi_)).

**Step 2 .**

A mayfly's velocity **v**=(*v*_1_,…,  *v*_*d*_) is initialized through its positional change. Its direction is decided as a hybrid interaction between individuals and the social flying experiences. To be specific, every mayfly tends to alter its trajectory in alignment with its personal best position (pbest) so far. It also alters based on the best position achieved by any other mayfly present in the swarm so far (gbest).

**Step 3 .**

The population of the male mayflies is initialized as *P*_Gmi_(*i*=1,2,…,  NG) with velocities *v*_*mi*_. The male mayflies, gathered in swarms, denote that the position of every mayfly gets altered in alignment with its individual's experience and that of the neighbor's. *P*_*Gi*_^*t*^ is assumed to be the current position of mayfly *i* in search space at time step *t* and the position gets altered with the addition of velocity *v*_*i*_^*t*+1^, to the current position. This notation is formulated as given herewith.

(11) $$\begin{array}{c} P_{\text{Gmi}}^{t+1}=P_{\text{Gmi}}^{t}+v_{i}^{t+1}. \end{array}$$

Male mayflies are considered as present a few meters above the water, with *P*_Gim_^0^ *U*(*P*_Gmmin_, *P*_Gmmax_), performing nuptial dance. It can be assumed that these mayflies lack great speeds due to constant movements. This results in the calculation of a male mayfly's velocity *i* as follows [38]:

(12) $$\begin{array}{c} v_{\text{ij}}^{t+1}=g*v_{\text{ij}}^{t}+a_{1}e^{-\beta r_{p}^{2}}\left({\text{pbest}}_{\text{ij}}-P_{\text{Gmij}}^{t}\right)+a_{2}e^{-\beta r_{g}^{2}}\left({\text{gbest}}_{j}-P_{\text{Gmij}}^{t}\right). \end{array}$$

Here, *v*_ij_^*t*^ corresponds to mayfly *i*'s velocity in dimension *j*=1,  ..., *n* at time step *t*, *P*_Gmij_^*t*^ denotes the mayfly's *i*^th^ position in dimension *j* at time step *t*, *a*_1_ and *a*_1_ correspond to positive attraction constants utilized in scaling up the contribution of cognitive and social components, respectively. Furthermore, pbest_*i*_ denotes the mayfly *i*^th^ best position which it had ever visited. Based on the minimization problems under consideration, the personal best position pbest_ij_ at the next time step *t*+1 is determined as given herewith.

(13) $$\begin{array}{c} {\text{pbest}}_{i}=\left\{\begin{array}{c} P_{\text{Gmi}}^{t+1},\text{iff}\left(P_{\text{Gmi}}^{t+1}\right)<f\left({\text{pbest}}_{i}\right)\\ \text{is kept the same, otherwise} \end{array}\right.\;. \end{array}$$

Following is the equation for the global best position gbest at time step *t*.

(14) $$\begin{array}{c} \text{gbest}\in \left\{{\text{pbest}}_{1},{\text{pbest}}_{2},\ldots ,{\text{pbest}}_{N},|f\left(\text{cbest}\right)\right\},\\ =\min\left\{f\left({\text{pbest}}_{1}\right),\;f\left({\text{pbest}}_{2}\right),\ldots ,f\left({\text{pbest}}_{\text{NG}}\right)\right\}. \end{array}$$

Here *β* represents the fixed visibility coefficient used in (7). It is utilized to confine the visibility of the mayfly to others. Further, *r*_*p*_ denotes the Cartesian distance between *P*_*Gi*_ and pbest_*i*_ and *r*_*g*_ corresponds to the Cartesian distance between *P*_Gi_ and gbest. Following is the equation used to determine these distances.

(15) $$\begin{array}{c} \left|\left|P_{\text{Gmi}}-X_{i}\right|\right|=\sqrt{\overset{n}{\underset{j=1}{\sum }}{\left(P_{\text{Gmij}}-X_{\text{ij}}\right)}^{2}}, \end{array}$$

where *P*_Gmij_ corresponds to the *j*^th^ element of mayfly *i* and *X*_*i*_ denotes the pbest_*i*_ or gbest. If the algorithm needs to function appropriately, then the best mayflies present in the swarm must continuously perform the up-and-down nuptial dance. So, the velocity of these best mayflies must be kept on changing which is calculated as follows [38]:

(16) $$\begin{array}{c} v_{\text{ij}}^{t+1}=v_{\text{ij}}^{t}+d\times r. \end{array}$$

Here, *d* denotes the coefficient of nuptial dance whereas the random value in the range of [−1, 1] is denoted by *r*.

**Step 4 .**

In this step, the female mayfly population is initialized *P*_Gfi_(*i*=1,2,…,  NG) with velocities *v*_*fi*_. Female mayflies tend not to gather as a swarm alike males. Instead, it tends to fly towards its male counterparts for mating. *P*_Gfi_^*t*^ is assumed as the current position of female mayfly *i* in search space at time step *t*, while its position gets altered with the addition of velocity *v*_*i*_^*t*+1^ to the current position, i.e.,

(17) $$\begin{array}{c} P_{\text{Gfi}}^{t+1}=P_{\text{Gfi}}^{t}+v_{i}^{t+1}. \end{array}$$

Here, due to *P*_Gfi_^0^ *U*(*P*_Gfmin_, *P*_Gfmax_) one cannot randomize the attraction process. So, the model is decided to be a deterministic process. As a result, their velocities are determined as given herewith in the presence of minimization problems [38].

(18) $$\begin{array}{c} v_{\text{ij}}^{t+1}=\left\{\begin{array}{c} g*v_{\text{ij}}^{t}+a_{2}e^{-{\beta \text{r}}_{\text{mf}}^{2}}\left(P_{\text{Gmij}}^{t}-P_{\text{Gfij}}^{t}\right),\text{ if }f\left(P_{\text{Gfi}}\right)>\;f\left(P_{\text{Gmi}}\right),\\ g*v_{\text{ij}}^{t}+fl\times r,\text{ if }f\left(P_{\text{Gfi}}\right)\leq \;f\left(P_{\text{Gmi}}\right). \end{array}\right. \end{array}$$

Here, *v*_*ij*_^*t*^ corresponds to the female mayfly's velocity *i* in dimension *j*=1,  ..., *n* at time step *t*, *P*_*Gfij*_^*t*^ denotes the female mayfly *i*'s position in dimension *j* at time step *t*, and *a*_2_ denotes the positive attraction constant whereas it remains a fixed visibility coefficient. Further, the gravity coefficient is denoted by *g*, and *r*_mf _corresponds to the Cartesian distance between male and female mayflies. Here *fl* corresponds to a random walk coefficient and *r* denotes the random value in the range of [−1, 1]. This value is determined based on (15).

**Step 5 .**

In this step, the Levy flight approach is involved in calculating the velocity of a mayfly candidate solution. Equation (19) is used to determine the velocity of the mayfly candidate solution [38].

(19) $$\begin{array}{c} v_{\text{ij}}^{t+1}=\left\{\begin{array}{c} V_{\text{max}}\text{,}\;\text{if }v_{\text{ij}}^{t+1}>V_{\text{max}},\\ -V_{\text{max}}\text{,}\;\text{if }v_{\text{ij}}^{t+1}<-V_{\text{max}}. \end{array}\right. \end{array}$$

This stage uses the Levy flight approach to alter the position of the global finest component. Though the Levy flight method has been used for exploration purposes so far, it is associated with a specific search.

Here, *V*_max_ is calculated as follows:

(20) $$\begin{array}{c} V_{\text{max}}=\text{Levy}\left(\lambda \right)*\left(P_{\text{Gmmax}}-P_{\text{Gmmin}}\right). \end{array}$$

Here, *δ*  corresponds to a scale factor designed in alignment with the search space element. The author fixed *δ* as 1.

(21) $$\begin{array}{c} \text{Levy}\left(\lambda \right)=0.01\;\frac{r_{5}\sigma }{{\left|r_{6}\right|}^{1/\beta }}. \end{array}$$

Further, *σ* is calculated as follows [42]:

(22) $$\begin{array}{c} \sigma ={\left[\frac{\Gamma \left(1+\lambda \right)\text{sin}\left(\pi \left(\lambda /2\right)\right)}{\left(\Gamma \left(\left(1+\lambda \right)/2\right)\lambda \left[2^{\left(\lambda -1\right)/2}\right]\right)}\right]}^{1/\lambda }. \end{array}$$

Here, Γ(x)=(*x* − 1)!, *r*_5_ corresponds to *r*_6_ indiscriminate numbers that lie in the range of [0, 1], and 1 < *β* ≤ 2, where there is a constant value, i.e., 1.5 incorporated for *β* in the current study [40–42].

Levy(*λ*) denotes the step length, incorporated by Levy distribution with infinite variance and mean values with 1 < *λ* < 3. *λ* corresponds to the distribution factor, whereas the gamma distribution function is denoted by Γ(.).

**Step 6 .**

Gravity coefficient value calculation [38]:

Gravity coefficient *g* value can be considered a fixed number that lies in (0, 1].

(23) $$\begin{array}{c} g=g_{\max}-\frac{g_{\max}-g_{\min}}{{\text{iter}}_{\max}}\times \text{iter}, \end{array}$$

where *g*_max_, *g*_min_ correspond to maximum and minimum values which can be taken for the gravity coefficient, and *iter* denotes the algorithm's current iteration, whereas the maximum count of iterations is denoted by iter_max_.

**Step 7 .**

Mayflies are mated and the offspring are evaluated.

The mating process between the mayflies is discussed by the crossover operator as given herewith. From the male and female population, each one parent is selected through the same selection process, i.e., the attraction of females towards the males. Specifically, fitness function-based or random selection of the parents can be made. In terms of the fitness function, the best female mates with the best male, the second-best female with the second-best male, etc. This crossover results in two offsprings for which the formulation is given herewith [38]:

(24) $$\begin{array}{c} \text{offspring}1=L\times \text{male}+\left(1-L\right)\times \text{female},\\ \text{offspring}2=L\times \text{female}+\left(1-L\right)\times \text{male}. \end{array}$$

Here, *male* denotes the male parent, and female corresponds to the female parent, while *L* is a random value within a specific range. The initial velocity of the offspring is fixed as zero.

### 3.1. The Pseudocode of the Improved Mayfly Algorithm

The pseudocode of the improved mayfly algorithm is devised as follows:

1. Formulate the objective function *f*(*C*_*T*_(*P*_Gi_)),  *P*_Gi_=(*P*_Gi1_,…,*P*_Gi*d*_)^*T*^
2. Set the male mayfly population *P*_Gmi_(*i*=1,2,…, NG) and velocities *v*_*mi*_
3. Set the female mayfly population *P*_Gfi_(*i*=1,2,…,NG) and velocities *v*_*fi*_
4. Evaluate solutions
5. Determine global best gbest
6. Do While stopping criteria are not meet
7.  Update velocities and solutions of males and females
8.  Evaluate solutions
9.  Rank the mayflies
10.  Apply Levy flight approach to evaluate the velocity of a mayfly candidate solution
11.  Determine the value of the gravity coefficient
12.  Mate the mayflies
13.  Evaluate offspring
14.  Separate offspring to male and female randomly
15.  Reinstate worst solutions with the best new ones
16.  Update pbest and gbest
17. End While
18. Post-process results and visualization

## 4. Results and Discussion

The current study used a microgrid model with three conventional generators, solar, and wind units. One of the generators is a combined heat and power generator, whereas the other two conventional generators are synchronous. As per [9], three conventional generators and their daily load profile details were used in the current study. During the improved mayfly optimization algorithm implementation, various parameters were chosen for the optimal search process.

In order to assess the proposed IMA, various scenarios were considered as given herewith.

- All sources included
- Thermal power generating units without renewable sources
- Thermal power generating units with wind source only
- Thermal power generating units with solar source only

### 4.1. Case 1: All Sources Included

**Figure 2:**
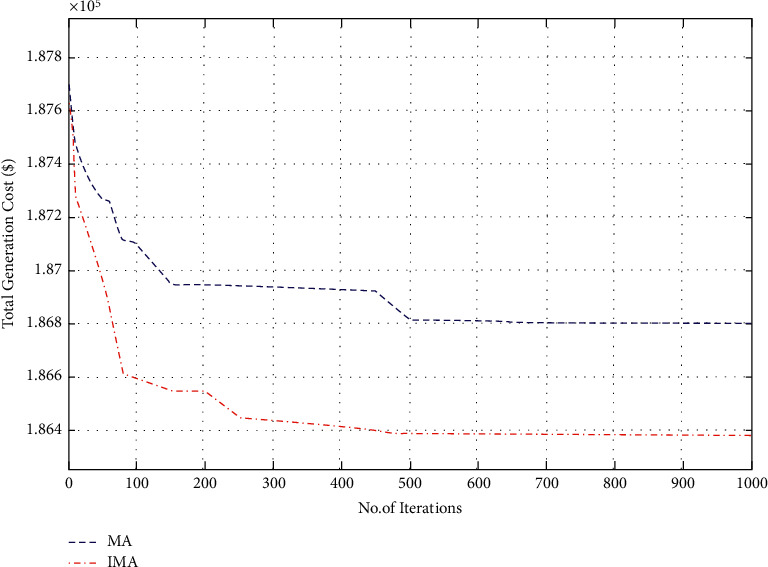
Convergence characteristics for total generation cost (case I).

**Figure 3:**
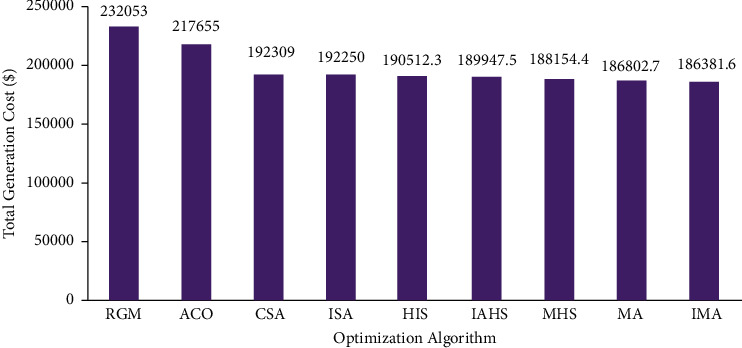
Comparison of total generation cost for case 1.

**Figure 4:**
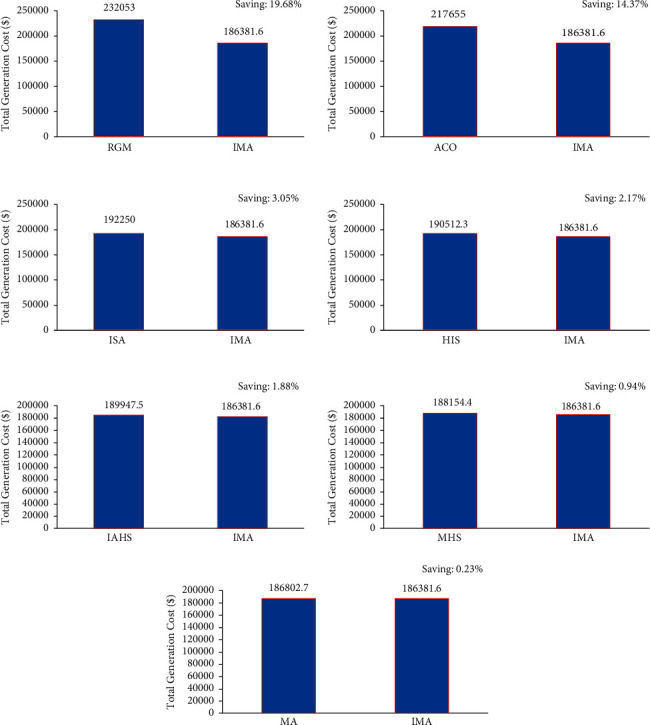
Total generation cost saving of CEED problem for case 1.

In the first case, the proposed IMO was utilized in the elucidation of the CEED problem in the microgrid and the case considered both wind and solar energy-powered generators. The optimization results were obtained using the proposed IMA and contrasted with the results obtained from other optimization algorithms. The generation cost calculated by the proposed IMA and other published methods is shown in Table 1 for comparative purposes.  Figure 2 shows the convergence characteristics required to mitigate the total generation cost incurred from MA and IMA algorithms. As shown in Figure 2, when cross-verifying the proposed algorithm's cost convergence characteristic, a quicker and more smooth transition was obtained than other optimization techniques considered. Further, Figure 3 shows the comparison results of total generation cost saving from CEED problem when using the mayfly algorithm and other such optimization algorithms. The results achieved from the simulation reveal that the proposed IMA is superior to MA and other optimization algorithms. Moreover, the total generation cost obtained using the proposed IMA algorithm was less than other optimization algorithms. In Figure 4, the total generation cost was obtained using IMA with MA and other published algorithms. It is observed from Figure 4 that improved mayfly optimization algorithm enhanced the total generation cost by 19.68%, 14.37%, 3.08%, 3.05%, 2.17%, 1.88%, 0.94%, and 0*.23*% over RGM, ACO, CSA, ISA, HIS, IAHS, MHS, and MA, respectively.

### 4.2. Case 2: Thermal Power Generating Units without Renewable Sources

**Figure 5:**
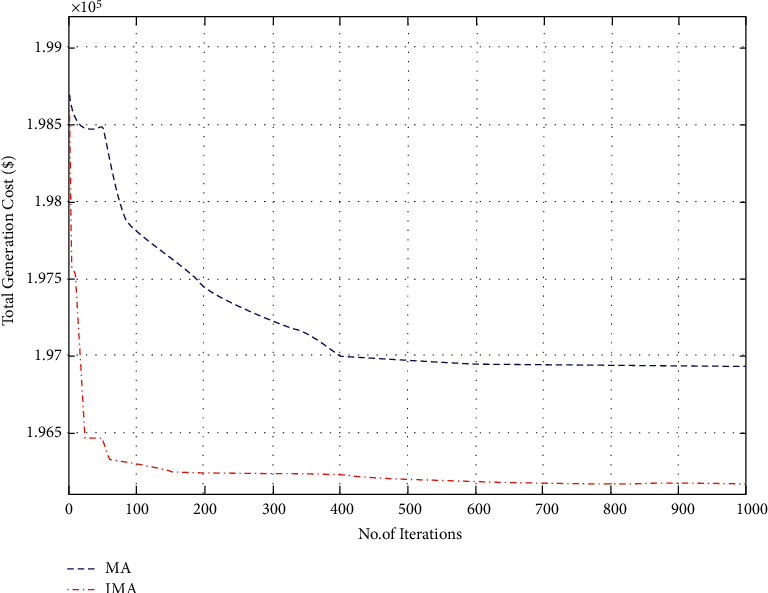
Convergence characteristics for total generation cost (case II).

**Figure 6:**
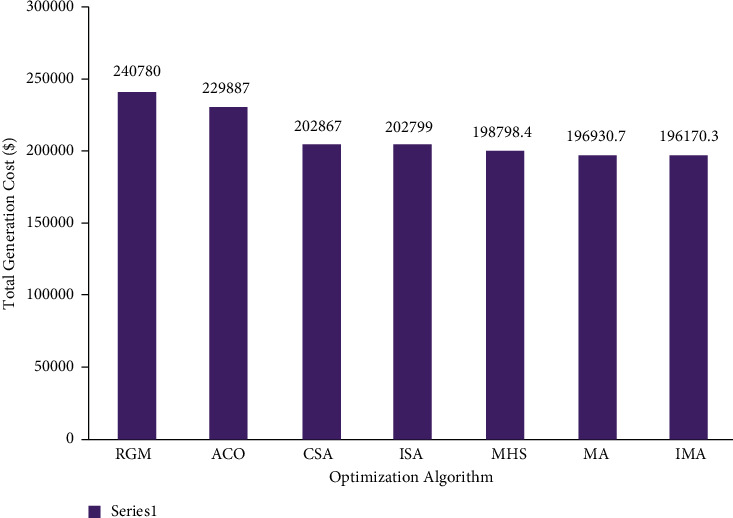
Comparison of total generation cost for case 2.

**Figure 7:**
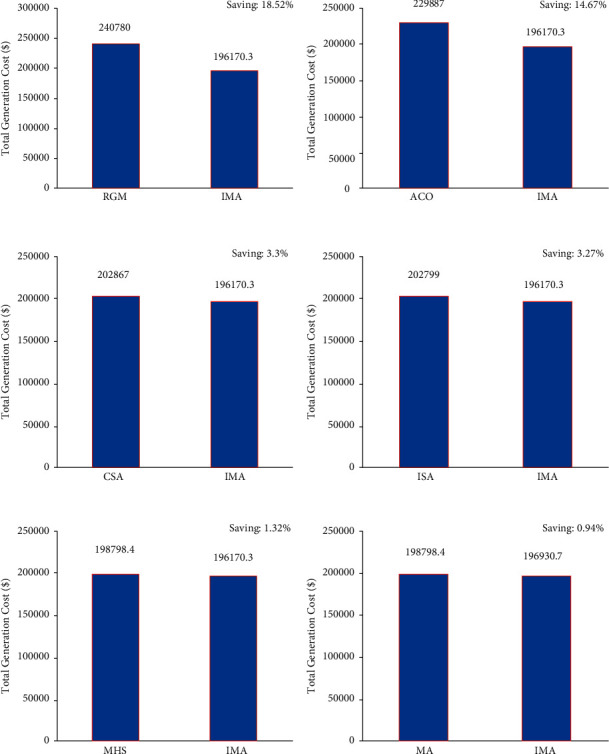
Total generation cost saving of CEED problem for case 2.

In this case, the mayfly optimization algorithm was utilized to resolve the CEED problem in the microgrid. The case took a total of 3 fossil-fuel-powered thermal generation units under consideration. The optimization results achieved by IMA were contrasted with other such algorithm results.  Table 2 shows the total generation cost achieved by the proposed IMA and other such optimization algorithms. The cost convergence profile results are shown in Figure 5 for the proposed IMA and other optimization algorithms. From the results, it can be understood that IMA has promptly converged to the optimal outcome. The comparison results of total generation cost savings are shown in Figure 6 for the CEED problem when using IMA. It shows that IMA achieved better results than MA and other optimization algorithms. The comparison of total generation cost savings obtained using MA and other published algorithms is presented in Figure 7. Figure 7 infers that the total generation cost improved when using the IMA algorithm by 18.52%, 14.67%, 2.93%, 3.3%, 3.27%, 1.32%, and 0.94% over RGM ACO, CSA, ISA, MHS, and MA, respectively. Furthermore, the total generation cost obtained in case 1 can be less than in case 2 due to incorporating renewable energy sources in a microgrid.

### 4.3. Case 3: Thermal Power Generating Units with Wind Sources Only

**Figure 8:**
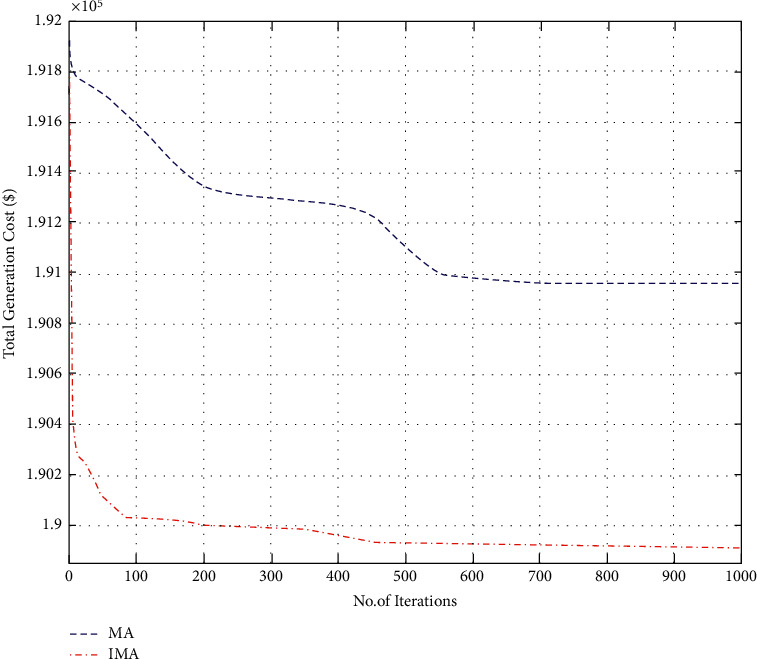
Convergence characteristics for total generation cost (case III).

Improved mayfly optimization algorithm was deployed in this case to resolve the CEED problem found in microgrids. This case considered fossil-fuel-powered thermal generators in addition to wind sources. IMA and other models (MA, IHS, CSA, and ISA algorithms) were simulated, and the results were compared.  Table 3 shows the generation cost calculated for IMA and other such optimization algorithms. In Figure 8, the author shows the cost convergence characteristic for the optimization algorithms under comparison and the proposed IMA.

**Figure 9:**
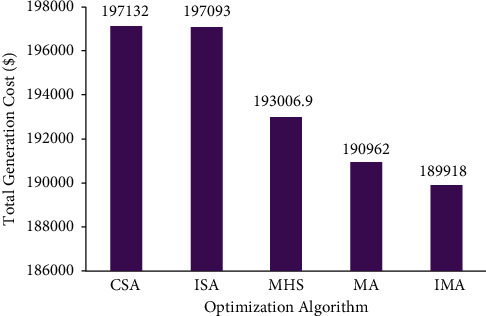
Comparison of total generation cost for case 3.

**Figure 10:**
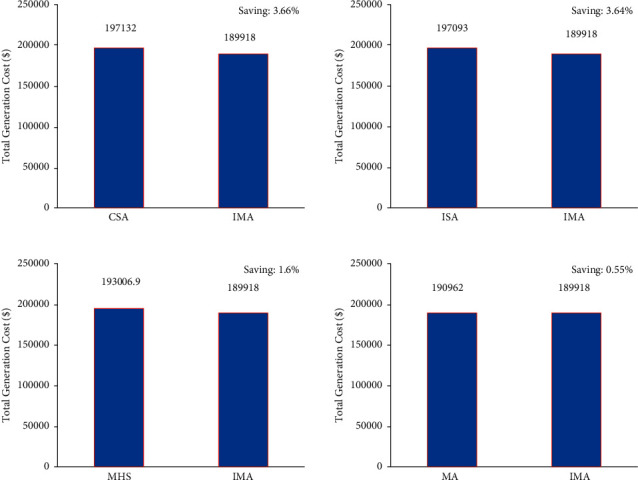
Total generation cost saving of CEED problem for case 3.

Further, Figure 8 also provides an inference; i.e., the convergence characteristic of the proposed LISA strategy II was smooth and quick compared to other strategies. In Figure 9, the researcher compared the total cost saving of IMA and other optimization algorithms from the CEED problem. It is observed from the application results that IMA yielded less total generation cost compared to MA, IHS, CSA, and ISA algorithms.  Figure 10 shows the comparison results of operation cost savings obtained using IMA and other published algorithms. It is observed from Figure 10 that IMA improved the operation cost by 3.66%, 3.64%, 1.6%, and 0.55% over CSA, ISA, MHS, and MA, respectively. Further, the total generation cost obtained in this case remains lower than in case 2 because of integrating a wind-powered energy source with the microgrid.

### 4.4. Case 4: Thermal Power Generating Units with Solar Source Only

**Figure 11:**
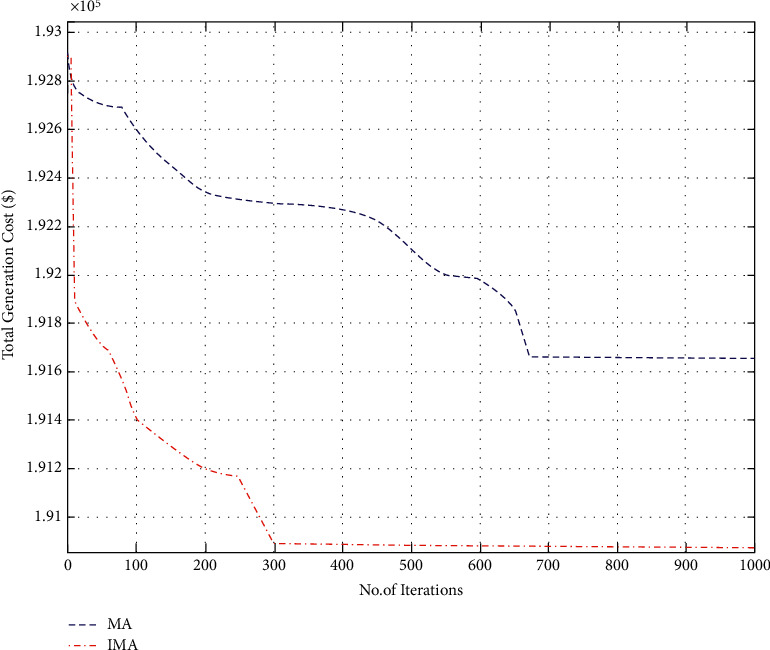
Convergence characteristics for total generation cost (case IV).

**Figure 12:**
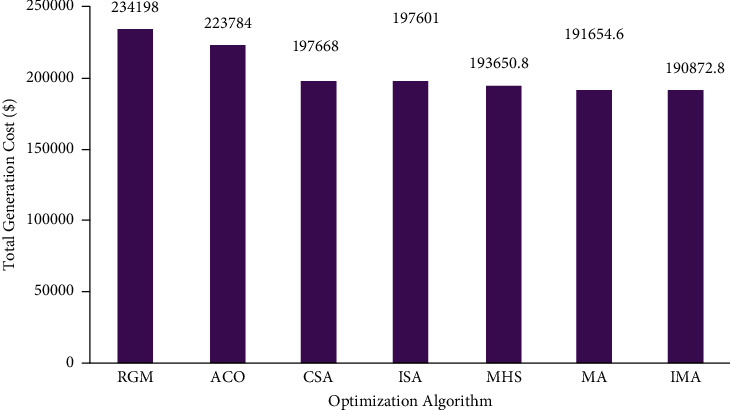
Comparison of total generation cost for case 4.

**Figure 13:**
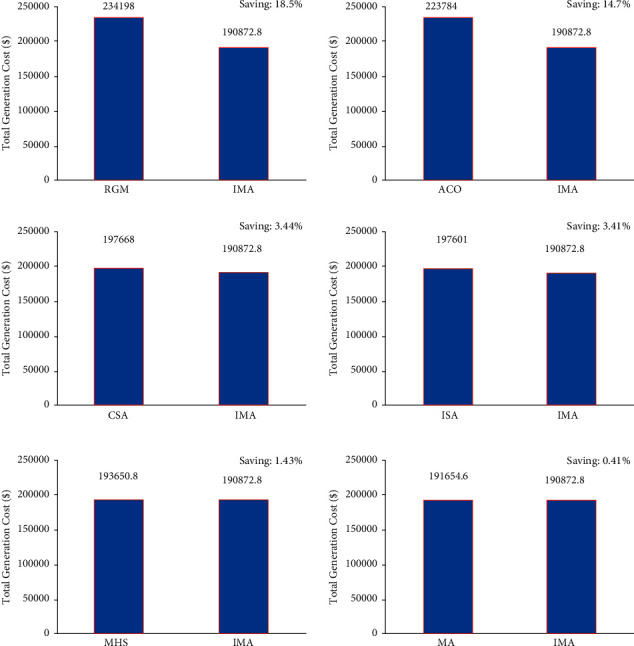
Total generation cost saving of CEED problem for case.

The case scenario considered fossil-fuel-powered thermal generating units with solar sources. In this scenario, improved mayfly optimization algorithm was selected to resolve the CEED problem found in microgrids. Simulation results obtained using an improved mayfly optimization algorithm are compared with the outcomes attained by MA and other such algorithms. The total generation cost of the improved mayfly optimization algorithm and other optimization algorithms is presented in Table 4.  Figure 11 represents the convergence characteristics obtained to minimize total generation cost using MA and IMA. From Figure 11, it is concluded that the proposed IMA provides steady and quick convergence characteristics.  Figure 12 show the comparison results of total generation cost saving achieved by IMA and other optimization algorithms for CEED problem found in microgrids. It is observed from the optimization results that an improved mayfly algorithm provides less total generation cost than other optimization techniques.  Figure 13 shows the optimization results of total generation cost saving obtained using IMA and other published metaheuristic optimization algorithms.  Figure 13 infers that the proposed IMA algorithm enhanced the total generation cost by 18.5%, 14.7%, 3.44%, 3.41%, 1.43%, and 0.41% over RGM, ACO, CSA, ISA, MHS, and MA, respectively. The authors also conclude that the total generation cost is less in this scenario than in case 2 because of the incorporation of solar-powered energy sources with the microgrid.

### 4.5. Comparison between the Cost Curves of All Scenarios

**Figure 14:**
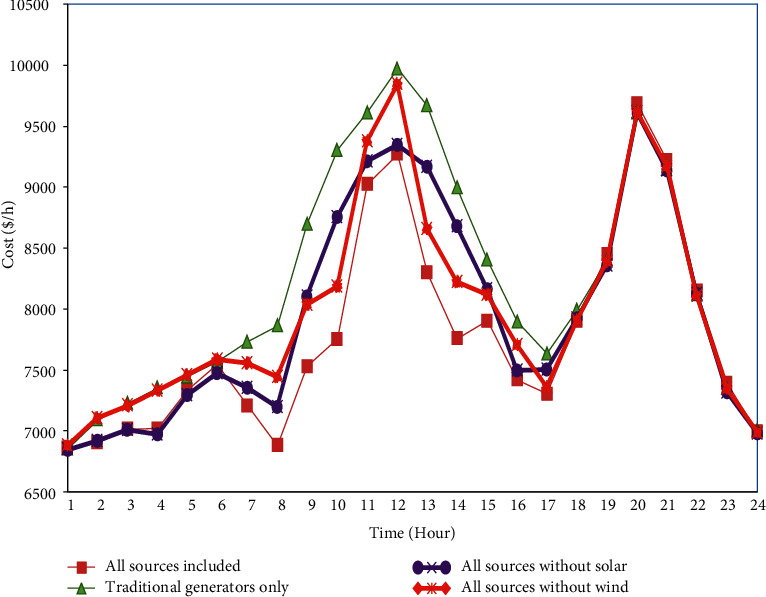
Comparison of the cost curve for all cases for 24 hours of a day using mayfly algorithm.

**Figure 15:**
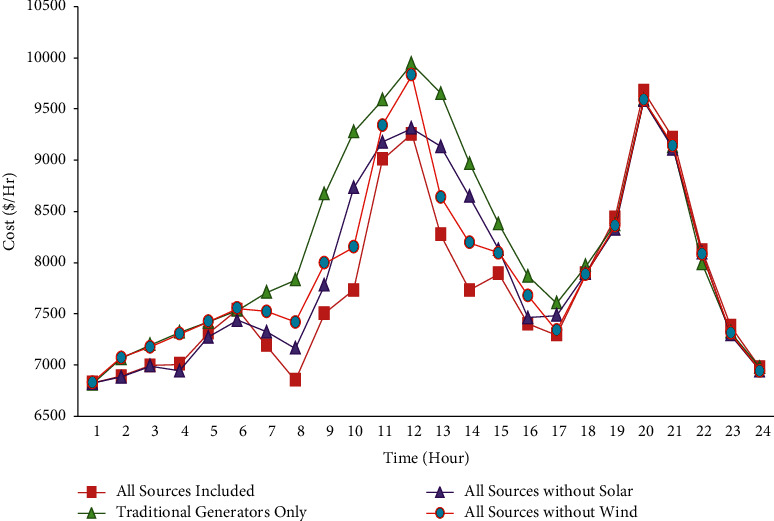
Comparison of the cost curve for all cases for 24 hours of a day using improved mayfly algorithm.

**Figure 16:**
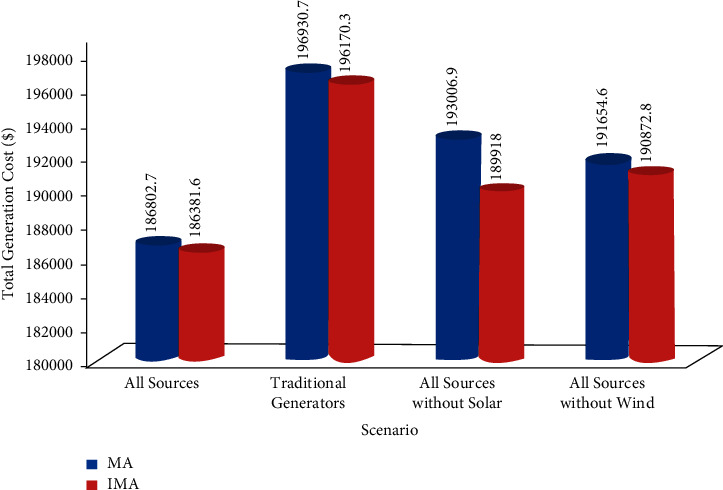
Comparison of total generation cost for all cases using MA and IMA algorithm.

Figures 14 and 15 show the comparison results of total generation cost curves under all the scenarios compared to IMA and MA algorithms 24 hours a day. Furthermore, Figure 16 shows the quantitative comparative results of total cost under all the scenarios using IMA. One can notice from Figures 14–16 that case 1 provides a minimum generation cost compared to other scenarios. Also, it can be observed from case 2 that the highest generation cost is obtained in this case. This might be attributed to the reason that renewable energy sources function as negative loads, while the rest are provided by the fossil-fuel-powered thermal generating units only. It reduces the total generation cost. Furthermore, the total generation cost obtained was less in case 3 than in case 4. It could have occurred due to heavy investment costs incurred upon solar power compared to wind power.

## 5. Conclusion

In the current study, the improved mayfly optimization algorithm (IMA) has been implemented to resolve the combined economic emission dispatch (CEED) with renewable energy sources. The study incorporated the proposed IMA as a solution for the CEED problem encountered in the microgrid. Solar and wind power are considered as the cost functions in this study. The proposed IMA algorithm was validated for its supremacy and efficiency in a microgrid model under varying scenarios. The outcomes of IMA and other algorithms were compared and contrasted. The comparison results show that the proposed IMA algorithm is better in cost reduction under all the scenarios. This infers that the proposed IMA is superior, robust, and efficient over other metaheuristic optimization algorithms published earlier. In future, the improved mayfly optimization algorithm can be applied to tackle the CEED problem in grid-connected microgrids comprising battery storage and electric vehicles to accomplish single and multiobjective optimization.

## Figures and Tables

**Table 1:** Optimal generation schedule of microgrid for case 1.

Time	RGM cost	ACO cost	CSA cost	ISA cost	IHS cost	IAHS cost	MHS cost	MA	IMA ($*/*h)
(h)	($*/*h) [5]	($*/*h) [5]	($*/*h) [5]	($*/*h) [5]	($*/*h) [9]	($*/*h) [9]	($*/*h) [9]	($*/*h)	
1	8529	7250	7153	7153	7090.5	7058.0	6942.8	6857.4	6824.6
2	8648	7511	7203	7203	7151.1	7130.2	7010.3	6904.7	6891.4
3	8675	7704	7278	7278	7170.8	7151.5	7100.7	7015.6	6994.8
4	8795	7742	7280	7285	7159.6	7130.8	7049.6	7020.3	7004.8
5	8758	8211	7545	7545	7528.2	7450.1	7377.2	7334.1	7309.7
6	8848	8459	7723	7679	7600.1	7572.2	7553.3	7544.2	7537.6
7	8964	8406	7457	7457	7444.2	7423.8	7294.1	7207.3	7189.5
8	9308	7923	7138	7138	7051.0	7050.3	6935.6	6879.7	6851.3
9	9609	9040	7731	7731	7660.3	7640.9	7576.4	7528.1	7505.8
10	10049	9599	7920	7937	7851.5	7845.4	7770.8	7752.6	7731.2
11	11520	11184	9231	9231	9152.0	9150.0	9073.4	9025.3	9006.8
12	12098	11616	9470	9470	9394.3	9381.3	9314.3	9271.4	9253.7
13	10676	10320	8482	8482	8400.3	8374.4	8326.2	8297.6	8273.9
14	9982	9707	8186	8186	8135.4	8119.9	8025.4	7758.9	7729.4
15	9569	9351	8154	8159	8100.6	8090.5	7984.4	7903.2	7892.7
16	9030	8469	7622	7626	7550.5	7539.6	7457.9	7419.7	7401.4
17	8872	8189	7526	7525	7470.6	7440.2	7362.6	7305.8	7291.3
18	9273	9061	8132	8131	8050.8	8040.4	7956.6	7904.3	7889.5
19	9990	9852	8652	8636	8549.6	8511.0	8462.3	8445.7	8436.1
20	12646	11897	9846	9811	9760.6	9710.0	9690.9	9681.4	9675.8
21	11496	11101	9383	9383	9249.9	9219.7	9221.6	9217.8	9216.1
22	9534	9488	8371	8370	8300.8	8281.4	8194.5	8146.7	8122.3
23	8667	8077	7572	7572	7463.7	7440.1	7403.1	7389.6	7378.4
24	8517	7498	7254	7262	7225.8	7195.7	7070.8	6991.3	6973.5
Total	232053	217655	192309	192250	190512.3	189947.5	188154.4	186802.7	186381.6

**Table 2:** Optimal generation schedule of microgrid for case 2.

Time	RGM cost	ACO cost	CSA cost	ISA cost	MHS	MA	IMA ($/h)
(h)	($*/*h) [5]	($*/*h) [5]	($*/*h) [5]	($*/*h) [5]	($*/*h) [9]	($/h)	
1	8490	7317	7179	7179	6977.4	6849.7	6810.2
2	8528	7694	7365	7367	7194.4	7089.6	7061.3
3	8592	7922	7479	7499	7310.3	7223.8	7198.5
4	8675	8117	7598	7608	7429.5	7351.7	7319.3
5	8756	8318	7721	7722	7550.8	7448.2	7416.7
6	8878	8600	7849	7851	7675.4	7567.1	7534.3
7	9005	8768	7978	7978	7802.5	7731.8	7707.4
8	9167	8998	8110	8110	7933.7	7861.8	7829.1
9	10527	10406	8943	8943	8774.3	8697.4	8669.1
10	11867	11347	9540	9540	9380.9	9304.8	9275.4
11	12664	12032	9851	9850	9696.6	9612.5	9590.1
12	13511	12476	10170	10170	10020.0	9973.4	9942.8
13	12664	12032	9850	9746	9696.6	9668.2	9651.6
14	11160	10889	9238	9230	9074.4	8994.6	8971.3
15	10009	9936	8657	8675	8483.5	8401.7	8375.4
16	9167	8998	8110	8109	7933.7	7894.6	7869.2
17	8875	8599	7849	7849	7675.6	7632.8	7605.2
18	9347	9186	8244	8244	8067.5	7992.7	7969.1
19	10009	9936	8657	8657	8483.5	8401.8	8372.4
20	12664	12032	9851	9847	9696.6	9613.8	9589.5
21	11495	11197	9388	9388	9226.1	9148.7	9129.2
22	9540	9479	8377	8379	8203.0	8115.3	7991.5
23	8675	8117	7598	7598	7429.5	7349.3	7317.6
24	8515	7491	7265	7260	7082.8	7005.4	6974.1
Total	240780	229887	202867	202799	198798.4	196930.7	196170.3

**Table 3:** Optimal generation schedule of microgrid for case 3.

Time	CSA cost	ISA cost	MHS	MA	IMA ($/h)
(h)	($*/*h) [5]	($*/*h) [5]	($/h) [9]	($/h)	
1	7153	7152	6943.4	6851.3	6819.6
2	7203	7199	7010.5	6917.4	6882.7
3	7279	7279	7099.6	7012.6	6990.8
4	7235	7235	7050.2	6972.8	6943.5
5	7544	7545	7377.2	7293.5	7269.3
6	7724	7724	7553.4	7476.8	7435.4
7	7606	7606	7439.4	7355.1	7321.9
8	7443	7443	7278.5	7194.3	7162.8
9	8364	8364	8190.1	8101.6	7784.2
10	9006	9006	8840.7	8753.4	8729.1
11	9454	9461	9295.8	9211.8	9178.6
12	9581	9581	9425.9	9346.9	9313.4
13	9408	9407	9248.7	9168.4	9132.8
14	8933	8933	8766.3	8679.5	8643.7
15	8427	8427	8252.3	8162.9	8123.1
16	7756	7758	7584.2	7495.4	7461.8
17	7761	7761	7590.1	7503.9	7481.6
18	8194	8193	8017.2	7927.1	7893.9
19	8636	8644	8461.9	8361.8	8329.2
20	9845	9842	9690.7	9613.9	9581.3
21	9383	9383	9221.8	9139.6	9107.9
22	8371	8325	8194.7	8127.4	8094.9
23	7572	7571	7403.7	7317.8	7293.5
24	7254	7254	7070.8	6976.7	6943.1
Total	197132	197093	193006.9	190962	189918

**Table 4:** Optimal generation schedule of microgrid for case 4.

Time	RGM cost	ACO cost	CSA cost	ISA cost	MHS	MA	IMA ($/h)
(h)	($*/*h) [5]	($*/*h) [5]	($*/*h) [5]	($*/*h) [5]	($/h) [9]	($/h)	
1	8490	7317	7179	7156	6977.4	6885.7	6827.4
2	8528	7694	7365	7364	7194.4	7106.1	7073.2
3	8592	7922	7479	7508	7310.3	7208.9	7174.3
4	8675	8117	7598	7599	7429.5	7335.2	7302.8
5	8756	8318	7721	7721	7550.8	7461.5	7423.9
6	8878	8600	7848	7841	7675.3	7589.8	7554.2
7	8849	8589	7816	7816	7647.2	7559.7	7523.4
8	8969	8559	7692	7692	7530.9	7447.5	7419.2
9	9788	9630	8269	8244	8105.9	8034.4	7994.2
10	10235	10139	8397	8337	8242.2	8187.3	8149.7
11	12153	11648	9620	9634	9465.9	9377.2	9341.8
12	13327	12336	10052	10053	9903.0	9847.2	9829.6
13	10957	10788	8887	8887	8734.9	8660.7	8639.1
14	10153	10012	8467	8467	8305.8	8227.1	8194.7
15	9707	9617	8377	8378	8206.8	8119.4	8094.9
16	9093	8829	7974	7970	7797.9	7707.6	7679.2
17	8810	8279	7608	7608	7444.4	7359.8	7336.2
18	9340	9137	8182	8182	8006.6	7912.4	7884.1
19	10009	9937	8657	8657	8483.5	8391.2	8357.4
20	12664	12032	9851	9849	9696.6	9617.2	9589.7
21	11495	11197	9388	9400	9226.1	9171.2	9143.8
22	9540	9479	8379	8379	8203.0	8112.3	8084.7
23	8675	8117	7598	7596	7429.5	7344.8	7312.2
24	8515	7491	7264	7263	7082.8	6990.4	6943.1
Total	234198	223784	197668	197601	193650.8	191654.6	190872.8

## Data Availability

The data used to support the findings of this study are included in the article. Should further data or information be required, these are available from the corresponding author upon request.

## List of Symbols

*a*: Annuitization coefficient
*C*_*w*_: Cost of wind power generating unit
*C*_*s*_: Cost of solar-powered generating unit
*d*: Coefficient of nuptial dance
*E*_*i*_(*P*_Gi_): Emission level of the *i*^th^ generator
*E*_*t*_: Overall emission
*fl*: Random walk coefficient
*F*_*i*_(*P*_Gi_): Fuel cost of the *i*^th^ generator
*F*_*t*_: Overall fuel cost incurred
*g*: Gravity coefficient
gbest: Global best
iter: Current iteration
iter_max_: Maximum no. of iterations
*L*: Random number
*l* ^*p*^: Costs incurred upon investment per unit installed power
*N*: Lifetime investment
NG: No. of generating units
*O* ^*E*^: Operating and maintenance costs per unit installed power
pbest: Personal best
*P*_Gi_^max^: Maximum output power of generator *i*
*P*_Gi_^min^: Minimum output power of generator *i*
*P*_*D*_: Total load demand
*P*_Gi_, *P*_Gmi_, *P*_Gfi_: Power output of *i*^th^ generating unit
*P*_*s*_: Output power from solar-powered generating unit
*P*_*w*_: Output power from wind power generating unit
*r*: Interest rate
*r*_*p*_: Cartesian distance between *x*_*i*_ and pbest_*i*_
*r*_*g*_: Cartesian distance between *x*_*i*_ and gbest
*r*_mf_: Cartesian distance between male and female mayflies
TC: Total operating cost
*β*: Fixed visibility coefficient
Λ: Price penalty factor
Λ_*i*_: Price penalty factor of *i*^th^ generator
Λ: Distribution factor
*δ*: Scaling factor
Γ(.): Gamma distribution function
*σ*: Standard deviation.

## Abbreviations

BSDE: Bernstein-search differential evolution
CEED: Combined economic emission dispatch
ChOA: Chimp optimization algorithm
CHP: Combined heat and power
CHPED: Combined heat and power economic dispatch
CNO: Collective neurodynamic optimization
DCHPED: Dynamic combined heat and power economic emission dispatch
DDG: Dispatchable distributed generator
DE: Differential evolution
DEED: Dynamic economic emission dispatch
DSM: Demand side management
ECED: Environment constrained economic dispatch
EED: Economic environmental dispatch
ELD: Economic load dispatch
EMA: Exchange market algorithm
FC: Fuel cell
FCDED: Fuel constrained dynamic economic dispatch
FP: Fractional programming
GA: Genetic algorithm
GWO: Grey wolf optimizer
HFA/HS: Hybrid Firefly and Harmony Search
IGWO: Improved grey wolf optimizer
IMA: Improved mayfly algorithm
ISA: Interior search algorithm
LPSP: Loss of probability of power supply
MA: Mayfly algorithm
MAACPSO: Modified adaptive accelerated particle swarm optimization
MBGSA: Memory-based gravitational search algorithm
MG: Microgrid
MILP: Mixed-integer linear programming
MOVCS: Multiobjective virus colony search
MT: Microturbine
PPF: Price penalty factor
PSO: Particle swarm optimization
PV: Photovoltaic
QPSO: Quantum particle swarm optimization
SFS: Stochastic fractal search algorithm
SGEO: Social group entropy optimization
SOS: Sequential optimization strategy
SSER: System surplus energy rates
WOA: Whale optimization algorithm
WT: Wind turbine.
